# Reactive Oxygen Species and Oxidative Stress in the Pathogenesis and Progression of Genetic Diseases of the Connective Tissue

**DOI:** 10.3390/antiox9101013

**Published:** 2020-10-19

**Authors:** Gustavo Egea, Francesc Jiménez-Altayó, Victoria Campuzano

**Affiliations:** 1 Department of Biomedical Science, University of Barcelona School of Medicine and Health Sciences, 08036 Barcelona, Spain; vcampuzanou@ub.edu; 2 Institut d’Investigacions Biomédiques August Pi i Sunyer (IDIBAPS), University of Barcelona, 08036 Barcelona, Spain; 3 Institut de Nanociencies I Nanotecnologia (IN2UB), University of Barcelona, 08028 Barcelona, Spain; 4 Departament of Pharmacology, Therapeutics, and Toxicology, Neuroscience Institute, Autonomous University of Barcelona, 08193 Barcelona, Spain; francesc.jimenez@uab.cat

**Keywords:** connective tissue, reactive oxigen species, oxidative stress, collagen, elastin, fibrilin, ground substance, collagen fibres, elastic fibres, genetic diseases

## Abstract

Connective tissue is known to provide structural and functional “glue” properties to other tissues. It contains cellular and molecular components that are arranged in several dynamic organizations. Connective tissue is the focus of numerous genetic and nongenetic diseases. Genetic diseases of the connective tissue are minority or rare, but no less important than the nongenetic diseases. Here we review the impact of reactive oxygen species (ROS) and oxidative stress on the onset and/or progression of diseases that directly affect connective tissue and have a genetic origin. It is important to consider that ROS and oxidative stress are not synonymous, although they are often closely linked. In a normal range, ROS have a relevant physiological role, whose levels result from a fine balance between ROS producers and ROS scavenge enzymatic systems. However, pathology arises or worsens when such balance is lost, like when ROS production is abnormally and constantly high and/or when ROS scavenge (enzymatic) systems are impaired. These concepts apply to numerous diseases, and connective tissue is no exception. We have organized this review around the two basic structural molecular components of connective tissue: The ground substance and fibers (collagen and elastic fibers).

## 1. Introduction

Connective tissue (CT) is the body’s structural support and a dynamic site for other important functions. For example, it is a medium for the exchange of metabolites; the defense, protection, and repair of the body; the storage and mobilization of energy (fat); the regulation and integration of mechanical and cell-signaling responses; the storage and mobilization of growth and differentiation factors; and a guide and barrier for cell locomotion and migration [1]. CT tightly interacts with other tissues to maintain functional organs. Most CTs originate from the mesoderm. From this embryonic layer, pluripotent mesenchymal cells are formed that migrate throughout the embryo, giving rise to adult CT cells, such as cartilage, bone, tendons, blood, and hematopoietic and lymphoid cells. CT is a major meeting point of metabolic and catabolic reactions of tissues and organs and a large platform of signaling that regulates them. One of the most general and significant processes is redox stress, which involves free radicals. Free radicals are by-products of a wide variety of physiological reactions that play crucial roles in living organisms at low concentrations. There is a fine equilibrium between their formation and degradation in tissues and organ systems, contributing to maintaining these species under nonpathological levels to preserve healthy homeostasis. However, the excessive accumulation of these mediators causes oxidative stress, which promotes, among other effects, CT injury.

This review addresses the involvement of oxidative stress in the pathogenesis and progression of connective tissue genetic diseases, of which most of them belong to the large family of rare diseases. Being “rare” does not mean that they have lesser importance in the clinical practice, and often their study reveals crucial physiological mechanisms occurring in health and other more common diseases. There are, indeed, other CT-associated diseases of nongenetic origin such as arthrosis, arthritis, and fibrosis, among others, in which ROS and oxidative stress also participate, but they are not going to be reviewed here because of space limitations.

### 1.1. Basic Structural Organization, Function, and Cellular and Molecular Components of Connective Tissue

CT is composed of cells and their surrounding extracellular matrix (ECM), which in turn consists of ground substance (proteoglycans, glycosaminoglycans (GAGs), and nonfibrotic/cell adhesion glycoproteins) and fibers (collagen and elastic fibers). Depending on the CT, cells, ground substance, or fibers are the predominant component and determine the histological classification. Fibroblasts are predominant in loose CT, fibers in tendons and ligaments, and ground substance in embryonic CT. Nonetheless, all three components are critical for the function of CT(s) in organs.

#### 1.1.1. Cellular Components

The CT is composed of resident and transient cellular components [2]. The most representative of the former group is the fibroblast [3]. Transient cells are those that (relatively) freely wander and move in and out of the tissue. Transient cells are almost exclusively represented by leukocytes and macrophages. Fibroblasts are the most abundant resident cell type of proper CT and are responsible for synthesizing almost all ECM components. Fibroblasts undergo different states of activity. Those that are highly active have an elongated morphology, with high transcriptomic activity. In contrast, when cells are scarcely active (called fibrocytes) they become smaller and have low transcriptomic activity. In both physiological states, cells are tightly associated with ground substance components and with collagen and elastic fibers (see below). Fibroblasts undergo cell division and restricted movement and can differentiate to other cell types such as adipocytes, osteoblasts, and myofibroblasts. In pathological circumstances, they can also be converted into epithelioid cells through the mesenchymal–epithelial transition (MET) mechanism. The reverse process, called epithelial–mesenchymal transition (EMT), also occurs and is relevant in cancer [4,5]. Myofibroblasts are modified fibroblasts that express some characteristic proteins of smooth muscle cells (SMCs) (some actin-based cytoskeleton proteins). Myofibroblasts acquire special relevance in wound healing and fibrotic processes [6].

#### 1.1.2. ECM Components

ECM is composed of a large variety of complex macromolecules localized in the extracellular space of the cells [7]. The extent of ECM varies with the tissue type. Cells maintain their associations with the ECM by forming specialized junctions that hold them to the surrounding macromolecules. ECM is not only the skeleton of tissues but also (1) modulates and determines the morphology and function of fixed and resident cells (see above), (2) influences their development and differentiation state, (3) regulates their migration and mitotic activity, (4) senses and transduces mechanical forces (compression and tensile) to cells, (5) facilitates junctional associations among cells, and (6) provides a biological field for immune defense. As indicated above, ECM is composed of a hydrated gel-like ground substance embedded with fibers. Ground substance resists compression forces and facilitates a quick exchange of metabolites and catabolites, whereas fibers support tensile forces.

##### Ground Substance

It is composed of GAGs, proteoglycans (GAGs linked to a protein core), and cell adhesive glycoproteins, also called nonfibrotic glycoproteins. GAGs are long, inflexible, unbranched polysaccharides composed of chains of repeating disaccharide units, an amino sugar (*N*-acetylglucosamine or *N*-acetylgalactosamine), and a uronic acid (iduronic or glucuronic) [8]. GAGs are classified into four groups, depending on their core disaccharide constituents [9]. GAGs are strongly negatively charged, attracting cations (such as K^+^, Na^+^), which, in turn, attract water that hydrates ECM and helps to resist compression forces. Unlike hyaluronic acid, GAGs are sulfated and usually consist of fewer than 300 repeating disaccharide units. The main sulfated GAGs include keratan sulfate, heparan sulfate, heparin, chondroitin 4-sulfate, chondroitin 6-sulfate, and dermatan sulfate. These GAGs are usually linked covalently to a core protein to form proteoglycans. An exception is hyaluronic acid that contains up to 10,000 repeating disaccharide units but does not form covalent links to some protein molecules. All GAGs are synthesized in the Golgi apparatus with the exception again of hyaluronic acid, which is synthesized as a polymer at the cytoplasmic face of the plasma membrane by hyaluronan synthases. Hyaluronic acid also has intracellular functions such as helping chromosome alignment during mitosis.

Proteoglycans are large structures that look like a bottle brush. They range from about 50,000 Da (decorin and betaglycan) to as large as 3 million Da (aggrecan) [9,10]. Aggrecan is responsible for the gel state of the ECM and acts as a barrier to fast diffusion of molecules [11]. Proteoglycans resist compression and can facilitate normal cellular locomotion of migrating cells to move between these hydrated macromolecules. At the same time, they can limit the migration of invasive microorganisms and metastatic cells. Proteoglycans also bind some signaling molecules and assist in the formation of collagen fibers (decorin). Syndecans are proteoglycans that remain attached to the cell membrane. Syndecans and betaglycans also act as low-affinity receptors (co-receptors) binding growth factors such as fibroblast growth factor (FGF) and tumor growth factor-beta (TGF-β), respectively, presenting them to their respective high-affinity receptors located in the vicinity at the plasma membrane. Moreover, they also act as hijackers of growth factors, regulating their availability for high-affinity signaling receptors [12,13,14,15].

Nonfiber glycoproteins or cell adhesive glycoproteins are also large macromolecules that have several domains in their 3D structure. At least one of the domains binds to the cell surface. The most representative of these glycoproteins are integrins, which bind to collagen fibers and proteoglycans [16,17]. In this manner, cell adhesion glycoproteins help cells to adhere to the extracellular matrix and hold various components of tissues to each other. Other major types of adhesive glycoproteins are fibronectin, laminin, entactin, tenascin, chondronectin, and osteonectin.

##### Fibers

Fibers of ECM provide tensile strength and elasticity. From the molecular perspective, there are only two types of fibers (collagenous and elastic), whereas from the histochemical point of view three types are defined (collagenous, reticular, and elastic fibers) [18].

##### Collagens

Collagen fibers are responsible for compressive forces, together with GAGs and proteoglycans. Collagen is a hard, inelastic glycoprotein that constitutes an abundant, large family of macromolecules with over 30 members. The most known and widely expressed are types I, II, III, IV, VII, VIII, IX, XI, XII, XV, and XVIII [19,20]. Collagen fiber is the result of the regular assembly of tropocollagen molecules, which are composed of three polypeptide α-chains. Alpha-chains are highly enriched in glycine, proline, hydroxyproline, and hydroxylysine, and each chain is coded by a single gene [21].

As indicated above, 30 types of collagens have been reported so far and they are grouped into four categories [22]. The first category is fibril-forming collagens, which have been taken as a model to report the basic structure and synthesis. Characteristic collagens of this group are types I, II, III, V, and XI [23]. The second category is fibril-associated collagens, which stabilize the previous group because they form molecular bridges between fibril-forming collagens and components of the ground substance. They are composed of types IX and XII. The third is network-forming collagens, which are synthesized by epithelial cells. As indicated above, they are not subjected to the action of procollagen peptidase and, consequently, form a network of thin 3D sheets. Examples are collagens IV (characteristic of basement membrane) and VII, which assist as anchoring fibers in the stabilization of the basement membrane. The fourth category is transmembrane collagens or collagen-like proteins, which are integral membrane proteins that participate in adhesion between tissues. This is the case of collagen type XVII that acts at the epidermis and the dermis, at the level of hemidesmosomes. Other collagens of this category are types XIII, XXIII, and XXV.

Collagens are synthetized in the ER by translation of the respective mRNA transcripts, generating a preprocollagen molecule whose proline and lysine residues are co-translationally hydroxylated by peptidyl proline and lysine hydroxylase, respectively [24]. Three preprocollagen molecules are aligned and assembled with the help of chaperones in the lumen of RER to form the procollagen molecule. Next, procollagen molecules leave the RER to the Golgi apparatus using large and pleomorphic transport carriers, where they are additionally glycosylated to be finally packaged in the *trans*-Golgi network and transported to the extracellular space. As procollagen is released to outside of the cell, extracellular plasma membrane-attached procollagen peptidases remove both amino and carboxyl ends of propeptides, resulting in a tropocollagen moiety. Tropocollagen molecules spontaneously self-assemble and align into a regular fibril array only in fibril-forming collagens. The resulting extracellular basic fibrillar structure of these collagens is subsequently thickened and stabilized (3D self-assembling) by covalent bonds between lysine and hydroxylysine residues of neighboring tropocollagen molecules by lysyl oxidases (LOXes) [25]. Importantly, the alignment of collagen fibrils and fiber bundles is determined by fibroblasts at the plasma membrane level, which act as a mold for the final correct direction of collagen fibrils. Subsequently, mechanical forces to cells will finally rearrange the orientation and organization of fibrils and bundles in the tissue [26]. Importantly, the aforementioned fibrillar structure for fibril-forming collagens is absent in types IV and VII collagen because propeptides are not removed from procollagen. In this case, the procollagen molecules assemble only into dimers, forming net-like structures.

##### Elastic Fibers

Elastic fibers provide most of the elasticity of CT. Elastic fibers are differently organized depending on the tissue to form long, worm-like, thin fibers, fenestrated sheets (the tunica media of large elastic arteries and internal elastic lamina of small arteries), or coarse bundles in dermis and elastic cartilage [27,28]. Fibroblasts and vascular SMCs (VSMCs) synthesize all components of elastic fibers.

Elastin is a glycoprotein that is rich in glycine, lysine, alanine, valine, proline, and desmosine (only in elastin) residues. However, unlike collagens, elastin does not contain hydroxylysine [29]. Like collagen, elastin comes from a soluble protein precursor, tropoelastin, which becomes insoluble because of cross-linking of lysine residues by LOXes [25]. Desmosine is highly deformable and, consequently, provides elasticity to elastic fibers, which explains cycles of stretching and recoiling. Elastin does not form elastic fibers unless the amorphous central elastin core is surrounded by a fibrillin-1 microfibril sheath [30]. During elastogenesis, besides fibrillin-1, several fibrillin-binding proteins facilitate the assembly of elastic fibers and their function, such as latent TGF-β binding proteins, fibulins, microfibril-associated glycoproteins (MAGPs), a disintegrin and metalloprotease with thrombospondin type-1 repeats (ADAMTS) and ADAMTS-like (ADAMTSL) proteins, and type VIII collagen, which limits the amount of stretching of elastic fibers. Transglutaminases and LOXes are also essential determinants of the final assembly and cross-linking of elastin and deposition onto microfibril scaffolds. Fibrillin-1 microfibrils interact with growth factors (TGF-βs and BMPs) and integrins. Fibrilin-1 mutations cause heritable connective tissue diseases, grouped as fibrillinopathies (see later). Several fibrillin-binding proteins have been reported. The most representative are latent TGFβ protein, fibulins 4 and 5, ADAMTS 6 and 10, ADAMTSL 2, aggrecan, MAGP-1 and 2, perlecan, aggecan, integrins αV, and LOX [31].

##### Other ECM Components

Finally, other parts of ECM are (1) the basement membrane, which forms the interface between epithelium and CT, and (2) integrins and dystroglycans, transmembrane glycoproteins that act as nonsignaling receptors of nonfibrillar/cell adhesive glycoproteins of the ECM and assist in the structure of basement membrane and CTs. Integrins function in adhesion and signal transduction from extracellular to intracellular media, activating second messengers at the focal adhesions.

### 1.2. Maintenance and Turnover of ECM

ECM is slowly but continuously (re)modelled for maintenance and adaptation to local homeostasis and pathological environments. Major components that are responsible for maintenance and turnover of ECM are a large family of proteases and their inhibitors, which are both secreted by fibroblasts, local and transient macrophages, some translocated leukocytes, and metastatic cells. Metalloproteases (MMPs), transmembrane inhibitors of proteases (TIMPS), soluble cathepsins, and other types of proteases belong to this group of ECM components [32,33,34,35].

### 1.3. Pathologies Associated with the Connective Tissue

As in any other tissue and organ, connective tissue is susceptible to damage, which is primary if it originates in some of the cell components or in any of the numerous ECM components and secondary because of alterations in any of the associated functions such as the metastatic process, immune overreactions, etc. In this section, we only review pathologies in which redox stress contributes to their evolution and that arise from damage in genes coding ECM components.

## 2. Redox and Oxidative Stress: Basic Concepts, Components, and Regulatory Pathways

### 2.1. Biochemistry of Free Radicals

Free radicals are atoms or molecules containing one or more unpaired electron(s) in the outer shell (valence shell). The unpaired electron(s) confers specific chemical properties on these molecules, such as the capacity to subtract electrons from other compounds to obtain stability [36]. However, this process transforms the molecule that loses its electron(s), so that it becomes a free radical itself, which may lead to modification of its own function and the function of other molecules. Additionally, molecules with unpaired electron(s) are short-lived and highly reactive because they are energetically unstable. Free radicals include molecules that are either positively or negatively charged or electrically neutral and may be organic or inorganic. Redox reactions involve oxidations and reductions. Oxidation means the gain of oxygen (O_2_) by a substance or the loss of an electron, while reduction mean the loss of O_2_, the gain of an electron, or the gain of hydrogen [37,38].

Reactive oxygen species (ROS) are small molecules formed by partial reduction of molecular O_2_, which participates in crucial biological processes such as cellular respiration and aerobic metabolism. However, O_2_ is a Janus-faced molecule, since reactive O_2_ intermediates are easily converted into toxic compounds that can cause cell damage through oxidation of proteins, lipids, carbohydrates, and nucleic acids. Singlet oxygen (^1^O_2_), O_2_^•−^, and H_2_O_2_ are the primary ROS products generated after the partial reduction of O_2_, while hydroxyl radical (OH^•^) and hypochlorous acid (HOCl) are generated in subsequent reactions [39]. O_2_^•−^ contains an unpaired electron and is a negatively charged species that does not diffuse across biological membranes. O_2_^•−^ may react rapidly with (1) Fe−S clusters, which can generate H_2_O_2_, (2) Fe^2+^, which can form OH^•^ by the Fenton reaction, and (3) superoxide dismutase (SOD), which dismutates O_2_^•−^ to form H_2_O_2_ and O_2_. On the other hand, the formation of HOCl and ^1^O_2_ can occur because of the catalytic activity of peroxidases (e.g., myeloperoxidase). The Haber–Weiss reaction, which includes the Fenton reaction, uses Fe^2+^ to generate the highly reactive oxidant OH^•^, when O_2_^•−^ and H_2_O_2_ are not metabolized. Notably, O_2_^•−^ can react with NO, at a diffusion-limited rate, to produce the highly reactive and harmful peroxynitrite (ONOO^•−^), which is either a nitrogen- or oxygen-centered radical species [40]. H_2_O_2_ is not a free radical. It is more stable than O_2_^•−^ and it can cross membranes through aquaporins [41]. H_2_O_2_ is produced constitutively in the mitochondria [42], in the membrane of the ER [43], and by NADPH oxidase NOX4 [44]. Other important sources of H_2_O_2_ result from the dismutation of O_2_^•−^ spontaneously or enzymatically via superoxide dismutase (SOD) [45].

### 2.2. Main Enzymatic Sources of Free Radicals

Endogenous free radicals are produced in environments of high O_2_ consumption, which mainly include intracellular organelles such as mitochondria, endoplasmic reticulum (ER), and peroxisomes. They are also produced in locations like the plasma membrane. The main endogenous enzymatic sources of ROS in mammals include (1) the mitochondrial respiratory chain, (2) cytochrome P450, (3) the flavoenzyme Ero1, (4) NADPH oxidases, (5) xanthine oxidase (XO), (6) lipoxygenases, (7) nitric oxide synthases (NOS), and (8) cyclooxygenases (COXes).

In the mitochondria, ATP is formed by oxidative phosphorylation, a process in which O_2_ is reduced to H_2_O along the electron transport chain. Under these circumstances, O_2_^•−^ is produced in several reactions catalyzed by enzymes in the internal membrane of mitochondria, where the greatest O_2_^•−^-generating capacity sites are complexes I and III [46]. Another source is the catalytic cycle of cytochrome P450, which metabolizes organic substrates principally via oxidations and involves the use of O_2_, giving rise to O_2_^•−^ and H_2_O_2_ as by-products [47]. Other enzymatic sources of mitochondrial ROS are NADH-cytochrome *β5* reductase [48], dihydroorotate dehydrogenase [49], complex II (succinate dehydrogenase) [50], and monoamine oxidases [51].

The ER also produces ROS because of protein-folding processes, NADPH oxidase enzymes, and flavoenzyme Ero1 activity. The last is an ER-resident oxidase responsible for disulfide bond formation to achieve oxidative folding of proteins [52]. In this process, O_2_ is consumed and H_2_O_2_ is produced as a by-product. When oxidative stress rises, it can increase Ca^2+^ leak from the ER lumen, which in turn stimulates mitochondrial ROS production [53].

The NOX family of catalytic subunits of NADPH oxidase are transmembrane-bound redox enzymes that represent the main source of ROS in vascular tissue, though they are also present in nonvascular tissues. The catalytic function of NOX isoforms is the reduction of O_2_ in the presence of NADPH to generate O_2_^•−^ and other ROS. In most mammals, the NOX family involves seven isoforms: NOX1–5, DUOX1, and DUOX2. All of them act as transmembrane catalytic subunits, but have different levels of action [54]. NOX-1–3 are activated by effector proteins (i.e., GTP Rac, NOXO1, NOXA1, p22^phox^, p40^phox^, p47^phox^ and p67^phox^) to assemble large functionally active complexes, while NOX4 is constitutively active and is regulated mainly by its level of expression by Poldip 2 [55]. NOX5 and DUOX1 and 2 are Ca^2+^-activated isoforms [56]. Unlike NOX3, the rest of the NOXes are all expressed in the cardiovascular system [57]. In addition, NOX5 is only expressed in human cells [58]. NOX isoforms localize to the plasma membrane, caveolae, endosomes, focal adhesions, ER, nucleus, and mitochondria [59]. Regarding the specific ROS produced by these enzymes, NOXes1 and 3 and NOX5 generate O_2_^•−^**,** while NOX4, DUOX1, and DUOX2 produce H_2_O_2_ [55]_._

On the other hand, XO is a soluble, membrane-bound O_2_^•−^- and H_2_O_2_-generating enzyme that plays a crucial role in the catabolism of purine nucleotides. It catalyzes the oxidation of hypoxanthine to xanthine and can further catalyze the oxidation of xanthine to uric acid (UA) [60]. Notably, when it accumulates, UA can be a pro-oxidant, but at physiological levels, it is the most potent non-enzymatic antioxidant in human plasma [61].

Other sources of ROS include lipoxygenases that catalyze the conversion of polyunsaturated fatty acids into leukotrienes and lipoxins, which mediate important cellular signaling pathways [62]. These enzymes generate O_2_^•−^ in the presence of reducing co-substrates [63]. NOS are the most important source of nitric oxide (NO) in biological systems. NO is a free radical and a potent vasodilator with many other relevant physiological functions. Three isoforms of NOS are known [64]: (1) Neuronal NOS (nNOS or NOS1), whose expression goes beyond neural tissue, (2) inducible NOS (iNOS or NOS2), whose expression is stimulated by inflammatory stimuli, and (3) endothelial NOS (eNOS or NOS3), predominantly located in endothelial cells and crucial to maintaining vascular homeostasis. These enzymes synthesize NO and use L-arginine as the substrate and O_2_ and NADPH as co-substrates. The eNOS/NOS3 also has the potential to generate O_2_^•−^ when some of its cofactors, tetrahydrobiopterin and L-arginine, are below physiological levels. This process is known as eNOS uncoupling [65]. Therefore, this phenomenon reduces NO synthesis and increases O_2_^•−^-formation, which in turn may scavenge NO to reduce its availability, leading to impaired NO-dependent relaxations and the formation of peroxynitrite (ONOO^•−^), an extremely toxic ROS that further exacerbate vascular injury.

Finally, COXes are the enzymes that generate prostanoids after oxidation of arachidonic acid, a polyunsaturated fatty acid present in the phospholipids. Two isoforms of COXes are reported: The constitutive isoform COX-1 and COX-2, which is generally induced by inflammatory stimuli and other mediators such as angiotensin II or endothelin-1 [66,67,68]. COXes generate ROS via oxidation of substances like NADPH [69] or their products (i.e., prostanoids), which may act as autocrine ROS stimulators [70].

### 2.3. Elimination of Free Radicals

Free radical levels are regulated by endogenous enzymatic and non-enzymatic antioxidant defense systems to prevent their accumulation and maintain cell redox homeostasis. Tripeptide glutathione, vitamins C and E, and UA provide non-enzymatic antioxidant mechanisms. However, antioxidant enzymes such as SOD, glutathione peroxidase, glutathione reductase, glutathione S-transferase, catalase (CAT), peroxiredoxins, and thioredoxin reductase are the most representative and provide the most specialized enzymatic antioxidant mechanisms in mammalian tissues.

There are three isoforms of SOD (SODs1–3) that dismutate O_2_^•−^ into H_2_O_2_ and O_2_ [71]_._ SOD1 (Cu/Zn) and SOD2 (Mn) are ubiquitous and, respectively, catalyze the dismutation of cytosolic and mitochondrial, or only mitochondrial, O_2_^•−^. Because it is secreted to extracellular spaces and anchored to the ECM, SOD3 (Cy/Zn) dismutates O_2_^•−^ in the extracellular space. SOD3 is highly expressed in vascular, lung, and kidney tissue. Catalase, glutathione peroxidase, thioredoxin reductase, and peroxiredoxin catalyze the removal of H_2_O_2_, which contributes to maintaining the equilibrium of this molecule [72]. Catalase, which is predominantly expressed in liver and erythrocyte peroxisomes in mammals, induces the breakdown of H_2_O_2_ to H_2_O and O_2_, but also H_2_O_2_ to H_2_O by oxidizing hydrogen-donating compounds [73]. Glutathione peroxidase and glutathione S-transferase reduce hydroperoxides, using glutathione as an electron donor. There are eight glutathione peroxidase isoforms with different tissue distribution [71]. Thioredoxin reductase catalyzes the reduction of thioredoxin using NADPH and participates in the reduction of hydroperoxides and in maintaining proteins in their reduced state [74]. Peroxiredoxins exist in six subfamily enzymes that are ubiquitously expressed. In general, these enzymes have peroxidase activity on peroxide substrates (e.g., H_2_O_2_, alkyl hydroperoxides, ONOO^•−^) using NADPH as the source of reducing equivalents and a thioredoxin system with the exception of peroxiredoxin 6, which uses glutathione peroxidase as the reductant [71,75].

Antioxidant response elements (AREs) are key components to cellular redox homeostasis in the reduction of oxidative stress episodes. The activation of these gene expression regulatory elements triggers fundamental antioxidant responses mediated by the expression of detoxification genes [76]. Multiple transcription factors interact with AREs to activate them and include nuclear factor erythroid 2-related factors 1, 2, and 3 (*Nrf1*, *Nrf2*, and *Nrf3*), small musculoaponeurotic fibrosarcoma proteins broad-complex, Tramtrack and Bric-a-brac, and cap’n’collar homology proteins, activating transcription factor 4, JUN proteins, and c-FOS and FRA proteins [76]. One of the most important transcription factors that combat oxidative stress through the activation of AREs is *Nrf2* [77,78]. KEAP1 is a repressor of NRF2 under homeostasis but, under stress conditions, NRF2 dissociates from KEAP1 and is translocated into the nucleus. This mechanism permits the binding of NRF2 to AREs, which leads to the regulation of gene expression of a wide repertoire of enzymes that metabolize oxidants, including glutathione S-transferase, NADPH dehydrogenase (quinone 1), SODs, peroxiredoxin, catalase, and glutathione peroxidase genes [79,80,81]. Notably, activation of the *Nrf2* pathway by exogenous compounds is possible [82] and can be potentially useful in the treatment of cardiovascular diseases [81,83].

### 2.4. Detection of Free Radicals

A wide variety of detection methods are available to measure free radical levels. These methods have advantages and disadvantages that depend on multiple factors and, thus, an exhaustive review was beyond the scope of this text. Here we briefly describe some of the most common techniques used to detect biomarkers of oxidative stress; for a more complete overview, the reader is referred to [84,85,86].

Free radical levels can be measured, among others, by chemiluminescent and fluorescent probes, chromatography methods, electrochemical biosensors, fluorescent proteins, spectrophotometry methods, and electron spins resonance [86]. It is worth noting that the results obtained by a single technique should be used with extreme caution and, whenever possible, validation by another technique and, whenever applicable, determination of the ROS-forming enzymatic source expression should be pursued. Lipid peroxidation is commonly used as a marker of oxidative stress because this process is involved in a variety of acute and chronic diseases. Malondialdehyde and *trans*-4-hydroxy-2-nonenal are routinely used as biomarkers of lipid peroxidation [87]. However, for example, the analysis of F2-isoprostanes levels is more robust because they are more stable molecules produced by nonenzymatic free radical-catalyzed peroxidation of arachidonic acid [88]. Tyrosine nitration is defined as the addition of a nitro group in the aromatic ring of tyrosine residues. Analysis of nitrotyrosine levels is often used as a measure of oxidative/nitrative stress, since nitrotyrosine is a relatively stable biomarker that correlates with disease activity and its levels decrease with therapeutic interventions. Commonly used techniques to measure nitrotyrosine levels include liquid chromatography, enzyme-linked immunosorbent assay, Western blot, and immunofluorescence [85]. Another form of stable oxidative modification of proteins is the formation of protein carbonyls, which are usually detected spectrophotometrically, or by enzyme-linked immunosorbent assay, Western blot, immunohistochemistry, or by high-performance liquid chromatography [85]. Dihydroethidium is a widely used fluorogenic probe to evaluate “in situ” oxidative stress production. Dihydroethidium is oxidized by numerous oxidants (e.g., O_2_^•−^, H_2_O_2_, ONOO^−^, OH^•^) to yield ethidium and 2-hydroxyethidium, which accumulate in cells and emit red (610 nm) fluorescence when interacting with DNA [89]. Interestingly, 2-hydroxyethidium constitutes a specific measure of O_2_^•−^-induced oxidation of dihydroethidium that can be measured by high-performance liquid chromatography [90].

## 3. Redox and Oxidative Stress in Genetic Diseases of Connective Tissue

We next discuss only the genetic diseases in which redox and oxidative stress has been reported so far. Table 1 summarizes each disease, its OMIN and ORPHAN numbers, the causative gene, and the radical species involved.

### 3.1. Genetic Diseases Affecting Collagen Fibers and Associated Components

Collagens are associated with a wide variety of diseases for which treatments are needed. Here, we provide a brief overview of recent progress in mechanisms of disease related to oxidative stress caused by mutations in collagens and the development of therapeutic strategies.

#### 3.1.1. Collagen IV-Associated Pathologies: Alport Syndrome

Alport syndrome (AS) is an inherited chronic kidney disease, characterized by nephritic symptoms that appear during early life and progressive impairment of renal function, leading to end-stage renal disease. Three distinct genetic forms of the disorder exist: (1) X-linked Alport syndrome, linked to mutations in the *COL4A5* gene, (2) autosomal recessive Alport syndrome with mutations in both alleles of *COL4A3* or *COL4A4* genes, and (3) autosomal dominant Alport syndrome also associated with heterozygous mutations in the *COL4A3* or *COL4A4* genes. Because *COL4A5* is located on the X chromosome, AS1 occurs more commonly in males and the condition usually progresses to end-stage renal disease by the age of 40 years [91]. However, the detailed mechanism of progression to end-stage renal disease has not been elucidated. Therefore, children or adults with AS have no specific treatment and the current therapy is the normalization of blood pressure and reduction of urine protein excretion to slow the rate of progression toward end-stage renal disease [92].

Few AS mouse models mimicking human clinical features have been developed [93,94,95]. Differences in the genetic background (e.g., C57BL/6 J or 129/Sv) are associated with different patterns of disease progression, which suggests that animal models are useful to elucidate the underlying mechanisms involved in the development and progression of the disease [96,97]. Furthermore, pharmacological therapy such as that with angiotensin-converting enzyme (ACE) inhibitors was shown to delay disease onset [98,99]. Importantly, various studies that take the mutant *Col4a*^−/−^ as a model of AS have shown the implication of oxidative stress in this pathology. Evidence of oxidative stress is demonstrated by a significant rise in the urinary heme oxygenase-1 (HO-1) and H_2_O_2_ excretion rate in the urine of Col4a3^−/−^ mice compared with age-matched wild-type controls [98,100]. Using dihydroethidium (DHE) staining as a marker of tissue ROS generation, Gomez et al. demonstrated that kidneys from *Col4a3*^–/–^ mice produce higher levels of mitochondrial ROS together with a high concentration of H_2_O_2_ in the urine [100]. In *Col4a3*^−/−^ hearts, oxidative stress was markedly elevated, including 50% reduction in the GSH:GSSG ratio, as well as reductions in the protein levels of the mitochondrial electron transport chain of complexes I, II, and IV and a 35% increase in malondialdehyde [101]. The results using RNA expression to compare the global transcriptome of whole kidney and hearts from *Col4a3*^−/−^ with littermate controls suggest that metabolic and mitochondrial dysfunction are major problems in AS mouse. Prominent among the downregulated genes in kidney were peroxisomal and mitochondrial fatty acid metabolism genes, such as *Acox2*, mitochondrial genes, such as *Pgc1* and the *Cyp450* gene family, and the antioxidant *Mpv17l* [100]. In hearts, the expression of *Hbb-b1, Alas2, Cnn1, Aqp7,* and *Ogdhl* genes was significantly reduced in *Col4a3*^−/−^ mice [101]. In addition, defective mitochondrial respiration has been observed in primary tubular cells and in cardiomyocytes isolated from *Col4a3*^−/−^ mice, as measured by oxygen flux analysis [102]. Electron microscopy images revealed stressed mitochondrial morphology in the Alport tubular renal cells and hearts [101,102]. In this context, two therapeutic approaches were taken with similar results. Treatment of *Col4a3*^−/−^ mice with anti-miR 21 that directly targets *Mpv17l* in kidney increases lifespan and protects *Col4a3*^−/−^ mice from kidney disease progression by preventing miR-21-mediated suppression of the PPARα fatty acid metabolism and mitochondrial biogenesis pathways and inhibition of mitochondrial ROS generation in the kidney [100]. Similarly, osteopontin deficiency improves renal function and mitochondrial respiration in the renal tubules, which reduces dynamin-3 expression [102] and the cardiac phenotype and myocardial mitochondrial respiration by rescue of 2-oxoglutarate dehydrogenase-like protein (OGHDL) expression [101].

#### 3.1.2. Collagen VI-Associated Myopathies: Bethlem Myopathy, Ullrich Congenital Muscular Dystrophy, and Myosclerosis Myopathy

Deficiency of collagen type VI (Col VI) caused by mutations of COL6 genes (*COL6A1*, *COL6A2,* and *COL6A3*) gives rise to three main muscle disorders: Bethlem myopathy (BM), Ullrich congenital muscular dystrophy (UCMD), and myosclerosis myopathy. BM is relatively mild with a later onset and displays a relatively mild and slowly progressive phenotype. UCMD is severe and shows diffuse wasting and weakness of skeletal muscles in the first year of life, associated with degeneration and regeneration of muscle fibers with more rapid progression of symptoms and premature death due to respiratory failure [103,104]. Myosclerosis is a nondystrophic myopathy characterized by early, progressive muscle and joint contractures that result in severe limitation of movement of axial, proximal, and distal joints, walking difficulties in early childhood, and toe walking. Muscle biopsy shows partial collagen VI deficiency at the myofiber basement membrane and absent collagen VI around most endomysial/perimysial capillaries [105].

Col VI myopathies share defective autophagy that impairs clearance of dysfunctional mitochondria [106,107] as well as mitochondrial dysfunction due to deregulation of the permeability transition pore (PTP), an inner membrane, high-conductance channel formed from dimers of the mitochondrial ATP synthase [108,109,110,111]. The mitochondrial defect has been identified in skeletal fibers and neurons of Col VI-null mice (*Col6a1*^−/−^) [108,112] and in myoblasts from UCMD and BM patients [113,114]. Mitochondrial monoamine oxidases (MAO), a ROS generator, is increased in *Col6a1* knock-out muscle. Not surprisingly, ROS production is higher in *Col6a1* knock-out muscle than control [115]. Additionally, suggesting a protective role for Col VI against age-induced oxidative damage, ROS production was significantly higher in the brain of aged *Col6a1*^−/−^ mice than in age-matched, wild-type samples, whereas younger mouse brains did not reveal any significant difference between the two genotypes [112].

Different therapies have been investigated for COL VI myopathies. Inhibition of cyclophilin D, which modulates the opening of the PTP in the mitochondrial inner membrane, reduces myofiber degradation and apoptosis in animal models of Col VI myopathy [110,116,117], cultured myoblasts [118], and muscle biopsies from patients with Col VI myopathy treated with cyclosporin A [119]. Myoblasts from patients, upon incubation with H_2_O_2_ or tyramine (MAO substrate), upregulate MAO-B expression and display a significant rise in ROS levels, with concomitant mitochondrial depolarization. MAO inhibition by pargyline significantly reduced both ROS accumulation and mitochondrial dysfunction. However, cyclosporine A could not prevent mitochondrial depolarization induced by tyramine, suggesting that MAO-dependent ROS accumulation is upstream of PTP opening, and that oxidative stress makes the latter event insensitive to cyclosporine A [120].

#### 3.1.3. Collagen VIII-Associated Pathologies: Fuchs Syndrome

Fuchs endothelial corneal dystrophy (FECD) is a progressive, bilateral condition characterized by dysfunction of the corneal epithelium, leading to reduced vision. The corneal endothelium is essential for maintaining the transparency of the cornea by regulating corneal hydration. Ultrastructural features of FECD include the loss of endothelial cells with thickening and excrescences of the underlying basement membrane (i.e., guttae), which are clinical hallmarks of FECD, becoming more numerous with the progression of the disease [121]. Genetic studies have identified multiple gene mutations and loci associated with FECD. Mutations positioned in the triple helical domain of collagen type 8 (*COL8A2*) alter the structure and composition of Descemet’s membrane, leading to the early onset of type I FECD [122,123,124]. Significant insights to understand Col 8 deposition in FECD and its relationship with young onset of the disease have been obtained due to the development of *Col8a1* knock-in [125,126] and knock-out [127] mouse models.

Corneal tissues from FECD patients display an overall increase of ROS, and human corneal endothelial cell lines derived from FECD patients are more vulnerable to oxidative insults (measured, among others techniques, by human oxidative stress and antioxidant defense RT-PCR arrays, high-sensitivity ELISA to quantify 8-hydroxy-2′-deoxyguanosine, and immunofluorescence) [128]. The corneal endothelial cells present an inefficient mitochondrial system including increased mitochondrial DNA damage, decreased mitochondrial membrane potential, and mitochondrial fragmentation [129,130]. Enzymatic antioxidants like SOD in cytosolic and mitochondrial forms, catalase, glutathione peroxidase, and glutathione reductase are also depleted in FECD [131]. Proteomic analysis of corneal endothelium from FECD patients showed specific downregulation of the peroxiredoxin family of antioxidants (*Prdx*1 and *Prdx*6) [132,133,134] and NRF2 [135].

In a therapeutic approach, researchers try to restore ATP production by stabilizing cardiolipin, a phospholipid present in the inner mitochondrial wall that is vulnerable to oxidative stress. Elamipretide, a synthetic mitochondria-targeted tetrapeptide that ameliorates mitochondrial dysfunction by preventing peroxidation of cardiolipin [136], is in phase II trials (Stealth Biotherapeutics ClinicalTrials.gov Identifier: NCT02653391).

Interestingly, in addition to oxidative stress and apoptosis that are indicated as the underlying mechanism for the progressive loss of endothelial cells in FECD [128], corneal samples from FECD [137,138] and knock-in mouse models [125] show upregulation of the unfolded protein response (UPR) evidenced by dilated ER, deregulated transcript levels of UPR markers (by PCR-array among the significant 42, GRP78, phosphoeIF2α, CHOP, EDEM3) [137,138]. A treatment strategy could be a combination therapy. Experiments to determine whether a reduction of ER stress could reduce dystrophic conditions and restore corneal transparency would provide insight into therapeutic strategy. For example, lithium, which can inhibit UPR and oxidative stress, promotes endothelial cell survival in the knock-in mouse model of FECD [139].

#### 3.1.4. Collagen XV-Associated Deficiencies

Genetic analyses have suggested that *COL15A1* is associated with atherosclerosis in aged individuals [140]. It can also act as a modifier of the severity of the thoracic aortic aneurysm [141]. It has a potential role in primary open-angle glaucoma [142] and has been implicated in Cuticular drusen, a subtype of age-related macular degeneration [143]. In the context of atherosclerosis, the expression of *Col15a1* in SMCs is interesting because COL15A1 affects both the proliferative and migratory phenotypes of this cell type. Thus, *Col15a1* knock-out in SMC markedly attenuated lesion formation by reducing SMC proliferation and impairing multiple proatherogenic inflammatory processes [144]. Other studies with knock-out mice have shown that collagen XV is important for the structure and function of microvessels in the striated muscle, heart, and skin [145,146]. Mice subjected to exercise-induced stress developed capillary rupture heart failure and muscle atrophy [145]. Under physiological conditions, these mice exhibited a reduced cardiac ejection fraction at 1 month of age, which was compensated at 5 months of age, by a still unknown mechanism [146]. Additional defects in *Col15a1*^−/−^ hearts included tortuous capillaries varying in thickness, frequent ruptures in the capillary walls, poor capillary perfusion, and abnormal extravasated erythrocytes [146]. In the skin of these mice, intravital microscopy revealed microvascular dysfunction including increased permeability, a decreased capillary perfusion index, reduced blood cell velocity, and lower microvascular blood flow rate [146]. Drosophila mutants of multiplexin (Mp), the orthologue of vertebrate collagen types XV and XVIII, exhibited morphological changes in cardiomyocytes and progressive dysfunction of the skeletal muscles, reminiscent of phenotypes observed in *Col15a1*-null mice [147]. Interestingly, Mp fly mutants showed morphologically altered mitochondria in indirect flight muscles, resulting in severely attenuated ATP production and enhanced ROS production. Mitochondria from Mp mutants showed abnormal cristae, swollen appearances, and diffuse outer membranes, which are signs of enhanced mitochondrial permeability due to mitochondrial PTP opening [108,148]. This suggests a pathomolecular mechanism shared with *COL6A1* mutations. Mitochondrial PTP opening is enhanced in mutants, and Mp collagens are required for mitochondrial homeostasis. The progressive phenotypes of MP-related diseases are attributable to mitochondrial dysfunctions [147]. Integrins mediating signaling from Mp to mitochondria are in accordance with the biochemical evidence that integrins engage in the regulation of mitochondrial ROS production by Rho GTPases and *Bcl-2* [149]. Mitochondrial dysfunction resulting from collagen VI or XV/XVIII deficiencies were ameliorated by cyclosporin A, an inhibitor of mitochondrial PTP opening or losartan, an angiotensin II type 1 receptor blocker. This suggests a potential convergent mechanism and treatment [119,147].

### 3.2. Genetic Diseases Affecting Elastic Fibers and Associated Components

#### 3.2.1. Elastin

The importance of elastin is highlighted by the variety of diseases caused by genetic alterations in the *ELN* gene with clinical consequences ranging from mild to life-threatening. These genetic alterations affect either the quantity or the quality of the deposited elastin and thereby affect the function of elastic tissues [150]. Most reported mutations within the *ELN* gene cause supravalvular aortic stenosis and autosomal dominant cutis laxa. More than 100 pathogenic or presumed pathogenic variants have been described in *ELN* to date in the literature, according to the ClinVar database (https://www.ncbi.nlm.nih.gov/clinvar) and the Human Gene Mutation Database (http://www.hgmd.cf.ac.uk). The most common genetic alterations that affect the elastin gene are large deletions that remove one copy of *ELN* in addition to the neighboring 25–27 genes as part of the recurrent microdeletion disorder Williams–Beuren syndrome (WBS) [151].

##### Supravalvular Aortic Stenosis

Supravalvular aortic stenosis (SVAS) is a heart defect that develops before birth (1:20,000 newborns). The condition is described as supravalvular because the section of the aorta that is narrowed is located just above the aortic valve. This narrowing usually makes it difficult for blood to leave the heart, which results in heart murmur and ventricular hypertrophy. Some people with SVAS also have defects in other blood vessels, most often stenosis of the pulmonary artery. If SVAS is not treated, the aortic narrowing usually leads to chest pain, shortness of breath, and, finally, to heart failure.

Most of the *ELN* gene mutations that cause SVAS result from a decrease in the production of tropoelastin [152]. Due to the shortage of tropoelastin, elastic fibers of the tunica media of the aorta become thinner. To compensate this, SMCs concomitantly increase in number (hyperplasia), resulting in a thicker aortic wall that narrows the lumen. A thickened aorta is less flexible and, therefore, less resistant to the stress of blood flow and pumping of the heart. Over time, there is a tendency to develop high blood pressure. The severity of SVAS, even among members of the same family, is highly variable. Strikingly, some affected individuals die in infancy, while others never experience symptoms of the disorder.

Notably, changes in oxidative stress seems to contribute to cardiovascular dysfunctions in individuals with elastin haploinsufficiency [153]. After a bioinformatic analysis of the quantitative trait locus peaks, *Ren1, Ncf1,* and *Nos1* significantly emerge as modifiers to predispose to hypertension and stiffer blood vessels [154]. Elevated renin in *Eln*-deficient mice has been described [155]. Renin is a major component of the renin-angiotensin pathway, whereas NO is important for influencing vascular tone. Both have known effects on blood pressure. Higher oxidative stress in the elastin-insufficient vessels has been correlated with *Ncf1* overexpression [154] *Ncf1* (encoding for p47phox) acts as a regulatory subunit for several NOX family members that are expressed in the vasculature [156,157], which contributes to the production of ROS.

Due to the dynamic and developmentally complex elastic fiber assembly, no therapy has been reported so far that can restore normal elastin to those with elastin insufficiency. Thus, the identification of genes that modify this pathology can serve as a basis for the identification of therapeutic strategies, which are mostly lacking in these types of pathologies.

##### Williams–Beuren Syndrome

Williams–Beuren syndrome (WBS) is a rare developmental disorder (1:10,000) with multisystemic manifestations caused by segmental aneusomy of 1.55–1.83 Mb at chromosomal band 7q11.23, which includes *ELN* and 25–27 additional genes (Williams–Beuren syndrome critical region, WBSCR). Besides the characteristic face and cognitive profile, the hallmark feature of WBS is a generalized narrowing of large elastic arteries, most notably SVAS, mainly due to *ELN* deficiency [158]. Histological characterization of arterial vessel walls of WBS patients shows increased number and disorganized elastic lamellar structures, fragmented elastic fibers, and hypertrophy of SMCs [159]. Arteriopathy is the main cause of morbidity in WBS, including systemic hypertension and other potential complications such as stroke, cardiac ischemia, and sudden death [160,161]. Differences in the WBS deletion that affect the copy number for *NCF1* finally affect hypertension risk on the severity of vascular stiffness [162,163]. Studies performed in *Ncf1* knock-out mice have revealed that p47^phox^ is one of the major effectors of Ang II [164], consequently Ang II-mediated oxidative stress in the vasculature was the proposed mechanism behind this protective effect in patients whose deletion includes a copy of *NCF1* [162,165].

The entire WBSCR is conserved in mice on chromosome band 5G2 in reverse orientation with respect to the centromere [166]. Two mouse strains were generated, each carrying half of the WBSCR deletion. According to their location with respect to the centromere, the two half deletions were named proximal deletion (PD, *Gtf2i* to *Limk1*) and distal deletion (DD, *Limk1* to *Trim50,* including *Eln*) [167]. DD mice presented with generalized arteriopathy, increased blood pressure, increased vessel stiffness, and cardiac hypertrophy [165,168]. Like humans, this cardiovascular phenotype has been associated with elevated AngII, increased oxidative stress markers, and *Ncf*1 expression. Treatments aimed at reducing NOX activity either via decreasing *Ncf1* gene dosage or pharmacologically with apocynin and losartan treatment both improved hormonal and biochemical parameters in DD mice, resulting in normalized blood pressure and improved cardiovascular histology [165].

A complete deletion (CD) model recapitulates the exact deletion observed in humans, in position and gene dosage [169]. The cardiovascular phenotype of CD mice is milder than that of DD mice [168,169], which suggests a modifying effect of gene(s) within or near the PD. Reduced expression of *Ncf1* was observed in affected tissues of CD mice [169,170]. Therefore, as in humans, *Ncf1* is likely to have an impact on blood pressure in this model.

Cardiac hypertrophy present in CD mice was associated with increased levels of oxidative stress in the heart due to dysfunction of the NFR2 pathway. Chronic administration of the antioxidant epigallocatechin-3-gallate (EGCG) rescues the hypertrophic cardiomyopathy, which restores nuclear levels of NRF2 in correlation with normalization of mRNA expression of target genes [170]. The mechanism by which ROS formation is augmented in the hypertrophic heart is currently unknown. Besides NADPH oxidases, there are several potential sources of superoxide anion formation, including uncoupled NOS and mitochondria. In this regard, ascending aortas from CD mice show the presence of luminal stenosis and compromised contractile responses to α1-adrenoceptor activation associated with increased NO signaling. The increased nNOS signaling may act as a physiological response against the detrimental effects of stenosis [171].

Recent studies also involve mitochondrial dysfunction in WS pathogenesis. In WS-derived primary fibroblasts, decreased basal respiration and maximal respiratory capacity was found, as well as increased ROS generation and decreased ATP synthesis [172]. This mitochondrial dysfunction could be due to loss of *DNAJC30*, a gene included in the WBSCR. DNAJC30 knock-out mice showed reduced ATP levels as well as alterations in mitochondrial function.

##### Cutis Laxa

Cutis laxa (CL) is a collection of disorders that are typified by loose and/or wrinkled skin that leads to a prematurely aged appearance. Many CL-related genes have been identified to date such as (1) genes in elastic fiber biogenesis (elastin), fibulin-4, fibulin-5, and latent TGFβ-binding protein 4, (2) genes required for intracellular protein trafficking (*ATP7A*, *ATP6V0A2,* and *RIN2*), and (3) genes required for cellular metabolism (*PYCR1, ALDH18A1,* and *SLC2A10*) [173]. Only mutations in *PYCR1* (ARCL2B) and *SLC2A10* ATS (see below) have been related with oxidative balance. The loss of *PYCR1* causes increased sensitivity to oxidative stress reflected by collapse of the filamentous mitochondrial network, decreased mitochondrial membrane potential, and a five-fold increase in cell death [174]. *SLC2A10*, which encodes GLUT10, was shown to transport dehydroascorbate (oxidized vitamin C) into mitochondria to limit the production of ROS [175]. *Slc2a10* knock-down in zebrafish produces disorganization of the vasculature, wavy notochord, and cardiac edema, as well as mitochondrial dysfunction and reduced TGF-β signaling [176]. Thus, *PYCR1* and *SLC2A10* would be required to maintain mitochondrial redox balance.

#### 3.2.2. Fibrillins and Fibrillin-Associated Proteins

Mutations in fibrillins (fibrillin-1 or fibrillin-2) lead to heritable connective tissue disorders known as fibrillinopathies such as Marfan syndrome (MFS), ectopia lentis (EL), Weill–Marchesani syndrome (WMS), MASS syndrome (Mitral valve prolapse, Aortic root diameter at upper limits of normal for body size, Stretch marks of the skin, and Skeletal conditions similar to Marfan syndrome), Shprintzen–Goldberg syndrome (SGS), and acromicric (AD) and geleophysic (GD) dysplasias. However, the molecular mechanisms that lead to their pathogenesis are less known, including the impact of ROS and redox stress.

##### Marfan Syndrome

Marfan syndrome (MFS) is an autosomal dominant negative disease with a prevalence of 1:5000, without gender or ethnic predisposition and affecting multiple organs and systems including cardiovascular, ocular, and skeletal ones. The most severe complications affect the cardiovascular system, including aortic root and ascending aorta aneurysms and dissections, and mitral valve regurgitation and prolapse. The lifespan of undiagnosed patients is around 40 years old, but this age is rather variable depending on the mutation occurring in fibrilin-1 gene (*FBN1*) and other unknown genetic and epigenetic determinants. The major ocular injury is *ectopia lentis* or the dislocation of crystalline lenses and myopia. The skeletal characteristics are rather evident and are reflected in high height (dolichostenomelia) due to overgrowth of long bones and longer and thin fingers (arachnodactyly). Additionally, patients can show pectus deformities (*pectus excavatum or pectus carinatum*), pes planus, and palate alterations [177,178]. Mutations in the *FBN1* gene are the cause of MFS and up to now almost 3000 mutations have been reported. Despite this large number, there is no correlation between the location or type of mutation and the resulting clinical phenotype. Depending on the *FBN1* mutation, the resulting mutant fibrillin-1 will cause a dominant-negative or haploinsufficiency effect in the disease, whose impact on the progress of the disease is poorly known, but it acquires potential relevance about the efficacy of current pharmacological treatments [179].

As indicated above, the fatal hallmark of MFS is aortic aneurysm, which usually ends with the dissection and rupture of the aorta. Whereas TGF-β has been postulated as an essential determinant in the pathogenesis of the aneurysm, its role has recently been questioned [180,181,182]. Nonetheless, it is now becoming clear that other molecular determinants significantly contribute to the aneurysm disorder, such as overproduction of ROS and the subsequent oxidative stress-associated damage to constituents of the aortic wall (tunica intima, media, and adventitia). Mice harboring mutations of *Fbn1* or *Fbn2* have provided significant insights into the understanding of microfibril-associated physiopathology. Nowadays there are several mice models bringing mutant forms of *Fbn1* [183,184], which lead to MFS (with several degrees of pathology). Through their use, it has been reported that (1) fibrillins-1 and -2 form copolymers and fibrillin-1 is mandatory for the postnatal maturation and mechanics of the aortic wall and (2) such copolymers regulate the availability of family members of TGF-β and BMP and other differentiation factors [185].

In human abdominal aortic aneurysms (AAA), dysregulated inflammation induction, MMPs’ activity, SMCs’ apoptosis and/or phenotypic switching, and ECM remodeling contribute to a variable extent to disease progression [186]. Nonetheless, the role of oxidative stress in AAA and in thoracic aortic aneurysm and dissection (TAAD) is less known. However, there is increasing evidence of its impact on the pathogenesis or progression of these conditions. Therefore, aortic aneurysms of genetic origin are also probably affected by oxidative stress.

TAAD of genetic origin involves different genes, which can be subdivided into three groups according to the (sub)cellular processes in which their encoded proteins are involved [187]: (1) ECM homeostasis (*COL1A1*, *COL3A1*, *COL5A1*, *LOX*, *MFAP5*, and *PLOD1*), (2) TGF-β signaling (*TGFB2*, *TGFB3*, *TGFBR1*, *TGFBR2*, *SMAD2*, *SMAD3*, and *SKI*), and (3) the SMC contractile apparatus (*ACTA2*, *MYH11*, *MYLK*, *PRKG1,* and *FOXE3*). To date, only *LOX*, *ACTA2*, *MYH11,* and *PRKG1* have been shown to be associated with oxidative stress.

*LOX* encodes lysyl oxidases (LOXes) and it is the only TAAD-related gene acting at the ECM that has been clearly linked to oxidative stress so far. LOXes are a group of ECM enzymes that initiate the formation of covalent cross-linkages between collagen and elastin, generating H_2_O_2_ as a by-product [188]. Experiments in *Lox*-deficient mice have shown that LOX-mediated cross-linking is essential for the maturation of the ECM, providing tensile strength [189,190]. Elevated LOX expression levels in a haploinsufficiency mouse MFS model (*FBN1^C1039G/+^*) correlated with the prevention of larger dilation of the aneurysm. Administration of LOX inhibitors blocked collagen accumulation and aggravated elastic fiber impairment, which initiated rapid progression of aneurysm dilatation [191]. Interestingly, LOX has also been identified as a novel vascular ROS source in hypertension. Thus, H_2_O_2_ produced because of LOX-induced cross-linking contributes to the pathogenesis of the disease [192]. However, it is unclear why LOX seems to offer a protective role in TAAD development yet causes oxidative stress in hypertension, whereas clinical manifestations of both diseases clearly differ. MFS patients are usually normotensive or even slightly hypotensive, while hypertension is characterized by high blood pressure levels, particularly diastolic blood pressure [193].

Other TAAD-associated genes regulate TGF-β signaling, which is a crucial signaling pathway for embryonic development, cell differentiation and proliferation, apoptosis, and ECM production and (re)modeling [194]. To date, deleterious effects in none of the TGF-β signaling-associated TAAD genes are known to cause excessive ROS production. Nevertheless, it is well-established that TGF-β signaling indirectly contributes to oxidative stress by stimulating ROS production and/or suppressing antioxidant systems in fibrosis, tumorigenesis, and cerebral ischemia [195,196]. The contribution of TGF-β-mediated oxidative stress has been demonstrated in MFS. This effect occurs through indirect regulation of the expression of NADPH oxidase NOX4 in MFS patients and mice [197] (see below).

Under normal circumstances, differentiated SMC expresses contractile-associated markers such as smooth muscle actin alpha 2 (*ACTA2*; encoding for α-SMA) and smooth muscle myosin heavy chain 11 (*MYH11*) [198,199]. Mutations in both of these genes are linked to TAA [187]. It is well known that vascular injuries are characterized by excessive production of ROS that, among other stimuli, can modulate SMC function and plasticity [200]. In such a pro-oxidant environment, contractile SMCs undergo a phenotypic switch toward a more synthetic fibroblast-like cell. This state is characterized by decreased expression of contractile-associated markers (e.g., α-SMA) and increased proliferation, migration, and ECM synthesis. It has been established that TGF-β signaling plays a dual role in SMC phenotypic switching in MFS [201]. However, the molecular mechanisms underlying oxidative stress-mediated SMC phenotype switching in TAA and MFS have not been elucidated. Oxidative stress can be both a cause and a consequence of loss of α-SMA. In vitro and ex vivo studies in human and mice aortic tissue could correlate excessive ROS production with increased expression of connective tissue growth factor (CTGF). Thus, oxidative stress regulates the SMC phenotype via CTGF [202]. NOX4 overexpression augments H_2_O_2_ levels, an effect that regulates both the differentiation of stem cells into VSMCs and the phenotypic changes between contractile and synthetic states [203]. ROS also switch VSMCs from a quiescent physiological contractile phenotype to a proliferative phenotype, which facilitates VSMCs’ migration, proliferation, and modification of the surrounding extracellular matrix [204]. VSMCs from p22phox-overexpressing mice exhibit increased H_2_O_2_ production and increased expression of synthetic phenotypic markers concomitantly with decreased contractile markers [205]. H_2_O_2_ also induces miR-145 expression in VSMCs to promote a contractile differentiation state [206], elevated ROS levels, and *NOX4* expression in isolated VSMCs and aortic tissue derived from *Acta*^−/−^ mice [207]. The loss of α-SMA favors the synthetic state, and ROS accumulation promotes NF-κB signaling, leading to increased expression of AngII receptor type 1a (AgRT1) [208]. Both TGF-β and AngII signaling phosphorylate Smad2 and Erk1/2 to initiate aneurysm formation. Consistently, losartan, an angiotensin receptor 1 inhibitor, prevented aortic aneurysm in MFS mice [209]. Of note, losartan, together with atenolol, is a pharmacological strategy given to MFS patients despite its demonstrated low efficiency in ameliorating aortic aneurysm [210,211]. A recent study in MFS mice has identified α-SMA as a possible redox stress target [197]. The α-SMA can undergo redox modifications (nitration and/or carboxylation), leading to impaired protein function. Thus, it can contribute to aneurysm formation and/or development. Therefore, regardless of whether α-SMA is mutated or not, oxidative stress seems to have a significant impact on SMC phenotype and function and, thus, on aneurysm formation and/or progression.

More recently, gain-of-function mutations in *PRKG1* have been associated with oxidative stress. *PRKG1* encodes for cGMP-dependent protein kinase 1, which is an essential mediator of VSMC tone through NO/cGMP-signaling. Here, basal protein kinase G (PKG) activity was significantly increased in mice carrying the *PRKG1* mutation, which leads to oxidative stress, increased VSMC apoptosis, and elastin fiber breaks [212].

Initial evidence for the contribution of oxidative stress in MFS came from a study examining endothelial function in MFS mice [213]. The endothelial-dependent relaxation in TAAD segments of *Fbn1^C1039G/+^* mice was severely affected because of the downregulation of eNOS/AKT signaling-induced NO. Subsequent preincubation of MFS aortic tissue with several ROS inhibitors improved acetylcholine (Ach)-induced aortic relaxation. Simultaneously, protein expression levels of ROS-producing enzymes XO, NOX, and iNOS increased with the concomitant reduction of SOD [214]. In another study, endothelial dysfunction was prevented in *Nox4*-deficient MFS mice [197], which involved, for the first time, NADPH oxidases in the progression of the MFS aortic aneurysm. In this study, MFS aortic tissue and cultured SMC derived from MFS patients showed NOX4 overexpression. In a newly generated MFS mouse model lacking *Nox4* gene expression, the integrity of elastic fibers was preserved and aortic aneurysm progression was significantly reduced. Remarkably, this finding was only significant in 9-month-old mice but not in younger mice. This suggests that NOX4 negatively influences aneurysm progression in later stages of the disease [197]. A similar protective role of Nox4 has been reported in cerebral arteries and aorta of Marfan mice and patients, respectively [215,216]. Altogether, an imbalance between ROS-producing proteins, including NOX4, and ROS-scavenging proteins actively impairs vasomotor function in MFS. Recently, besides eNOS, iNOS has been involved in MFS aneurysm formation [217]. This group reported increased iNOS levels in MFS mice and human aortic tissue, which were reverted after the administration of an iNOS inhibitor quickly normalized aortic size.

Ex vivo experiments using *Fbn1^C1039G/+^* aortic tissue samples showed that imbalanced production of COX-derived prostanoids, especially COX-2, also contributes to vasomotor dysfunction in MFS [218]. Moreover, the administration of a nonselective COX-inhibitor, indomethacin, to the hypomorphic mouse MFS model (*Fbn1^mgR/mgR^*) efficiently attenuated elastin degeneration, inhibited macrophage infiltration, and reduced MMP-2 and MMP-9 overexpression [219]. Interestingly, the contribution of COX-2 in MFS (*Fbn1^C1039G/+^*)-induced aortic dysfunction was sex dependent, since aortic anomalies of this enzyme were only detected in males [220]. These findings show that COX-2-derived prostanoids influence vasomotor aortic function in MFS.

The role of oxidative stress in the pathophysiology of vascular alterations in MFS is becoming clearer. Pioneer studies showed that FBN1 mutation in MFS mice (*Fbn1^mgR/mgR^)* was related to increased ROS production together with increased TGF-β and p38 MAPK signaling [221]. Shortly afterwards, elevated oxidative stress levels in plasma and aortic homogenates (pooled ascending aorta and aortic arch) were reported in *Fbn1^C1039G/+^* mice [214] and MFS patients [222]. More recently, a study demonstrated that ROS levels are exclusively increased in the dilated segments of the aorta. Thus, ROS enhancement was only present in the ascending aorta of *Fbn1^C1039G/+^* mutants and not in the descending arm [223]. These results agree with the different impact that MFS produces in the aortic reactivity of *Fbn1^C1039G/+^* mice, in which it induces either increased or decreased α1 adrenergic contractions in ascending and descending thoracic aorta, respectively [197]. Active phosphorylated forms of SMAD2 and Erk1/2 only increased in the affected segments [180,224]. These results reinforce the postulate that an interplay between TGF-β and ROS contribute to TAAD development. However, we must bear in mind that different mice models of MFS might provide conflicting results. This is the case of MFS mice (mgΔ^loxPneo^), a MFS mouse model in which *Fbn1* exons 19–24 were replaced by a neomycin-resistant expression cassette [225]. Authors reported that, whereas ROS were enhanced in later stages of aortic dilation, ROS reduction with lipoic acid did not prevent aortic dilation and elastic fiber injuries. Therefore, oxidative stress was uncoupled from aortic wall injuries [226]. In *Fbn1*^C1039G/+^ mice, ROS inhibition with apocynin (an unspecific NADPH oxidase inhibitor) attenuated aortic aneurysm progression and AngII-dependent enhanced ROS production in a TGF-β-dependent manner [223].

Oxidative stress comes not only from sustained increases in ROS production over time, but also from the reduced activity of scavengers from which glutathione is the main system. In aortic tissue from MFS patients, it has been reported that reduced activity of glutathione-S-transferase and glutathione peroxidase occurs, in conjunction with a decrease of reduced glutathione [227]. Therefore, the depletion of scavengers with or without increases of ROS generation could aggravate the aortic aneurysm in MFS.

Mitochondrial-derived increased ROS production has recently been associated with cell senescence. Aortic tissue and SMC isolated from MFS patients show accelerated senescence. This effect is at least partly mediated by ROS-induced activation of NF-κB signaling [228].

Other important sources of ROS in the cardiovascular system are XO, NO, and COXes. XO links purine metabolism to redox signaling and stress. UA and superoxide anion are the two main products from XO activity. Elevated levels of serum UA in humans are often associated with an increased risk of cardiovascular disease [229]. It is important to bear in mind that UA in physiological serum concentrations acts as a powerful antioxidant in the blood by scavenging ROS [61,230]. UA accounts for more than 50% of the total antioxidant capacity of biological fluids in humans [231]. UA was found in the wall of human aortic aneurysms and atherosclerotic arteries [232]. These findings suggest that UA might aggravate or attenuate the formation and/or progression of aortic aneurysms, including those present in MFS [230]. Nevertheless, further research is needed to elucidate the exact role of UA in MFS, as it is currently unknown whether it acts as an antioxidant or pro-oxidant.

Numerous therapeutic strategies are being investigated to fight against TAAD [233]. Therapies based on antioxidants have shown relative success, most probably due to the complexity of multiple pathways that tightly regulate the balance between ROS production and scavenger systems. In any case, their use deserves further attention considering recent results. This is the case of cobinamide, an analog of the free radical-neutralizing vitamin B_12_, which prevented aortic wall degeneration in a heterozygous mutant mouse for protein kinase G1 (PRKG1) that leads to its overactivation. The antioxidant N-acetylcysteine also ameliorated the aortopathy in these mutant mice [212]. Another promising example comes from the use of resveratrol in MFS [234]. Resveratrol is a potent polyphenol that is present in high concentrations in plants, nuts, and the skin of grapes. Treatment of MFS mice (*Fbn1^C1039G/+^*) with resveratrol reduced NOX4 expression and MMP2 activity and changed eNOS/iNOS and miR21/miR29 balances, which improved SMC survival. Moreover, resveratrol improved cardiomyocyte homeostasis, which probably activates mitochondrial sirtuin1 (SIRT)1 and increases SOD expression [235].

##### Weill–Marchesani Syndrome

Fibrillin-1 is composed of individual domains like multiple tandem arrays of epidermal growth factor-like domains (EGF-like domains) and cysteine-containing domains (TB domains). An RGD (arg-gly-asp) motif that binds integrins is present in TB4 domain [236,237] and adjacent TB5 binds heparin [238]. Different mutations in the TB5 domain cause autosomal dominant Weill–Marchesani syndrome (WMS) or acromicric (AD) and geleophysic (GD) dysplasias [239]. In contrast to MFS, these cause short stature, thickened skin, joint defects, and ocular problems. Unfortunately, very little is known about the contribution of ROS and oxidative stress in these diseases. Nevertheless, in a small cohort of WMS patients, plasma levels of lipid peroxide (LPO), TNF-α, and NO were elevated with a concomitant reduction of antioxidant capabilities. This suggests that redox dysfunctions contribute to the pathogenesis of WMS and point to antioxidants and free radical scavengers as a potential therapy to ameliorate the disease [240].

##### Systemic Sclerosis

Tissue fibrosis is the hallmark of systemic sclerosis (SSc) and the uncontrolled wound-healing process. Systemic sclerosis (SSc) or scleroderma is a chronic autoimmune disease characterized by tissue fibrosis and immune abnormalities, and the most common form of acquired scleroderma [241]. It is characterized by progressive thickening and hardening of skin and multiple internal organs. Inflammatory infiltrates and fibrosis of blood vessels in the dermis precede human SSc. *Tsk/+* (*tight-skin*) mice have evidenced dysfunctions of fibrillin-1 microfibrils in SSc in the dermis, particularly fibrillin-1 aggregates and fragmented elastic fibers [242]. It is well known that oxidative stress linked to vascular injury plays an important role in the pathogenesis of SSc [243,244,245,246,247]. Circulating levels of ROS and related markers correlate with SSc vasculopathy, fibrosis onset, and autoantibodies’ production [248]. The excess of ROS stimulates endothelial injury and other vascular alterations, which activate TGF-β-mediated EMT, a process that converts endothelial cells into myofibroblasts [249]. ROS cause chemical modifications in some lipids, proteins, and nucleic acids [250], which generate new epitopes that induce strong autoimmune responses. This is what has been shown in SSc, with ROS-associated changes in gene expression pattern and the stimulated release of IL-6, IL-8, and IL17 among others [251,252]. In this respect, diverse therapeutic strategies to interfere with ILs’ expression and/or their release have been successfully addressed with drugs such as the phosphodiesterase type 5 inhibitor sildenafil [253], the natural flavonoid kaempferol [254], EGCG [255], and the anti-IL-6 receptor antibody tocilizumab [256], among other treatments [257]. Many of these treatments also have an antioxidative stress effect that reduces or prevents the characteristic abnormal accumulation of ROS in this disease [258]. Other antioxidants, such as hydrogen sulfide, have been reported to interfere in the onset and progression of this disease [259]. NRF2, a transcription factor that induces the transcription of antioxidant genes (for example *GSH*) is downregulated in cultured skin fibroblasts derived from SSC patients. This observation was confirmed in both skin and lungs of SSc mice. Treatment with the NRF2 agonist dimethyl fumarate (DMF) reduced fibrosis and immune overactivation [260]. One of the main sources of ROS in SSC are NADPH oxidases [261,262]. Increased NOX2 and NOX4 have been reported in SSC fibroblasts, neutrophils, monocytes, and T lymphocytes [263,264,265]. In addition, NOX4 is highly expressed in skin of SSc patients and in cultured SSc fibroblasts. This overexpression is triggered by TGF-β via PKCδ and Smad2/3 [266].

##### Loeys–Dietz Syndrome

Loeys–Dietz syndrome (LDS) is not a disorder caused by mutations in CT structural components, but CT is severely affected because mutations in the TGF-β receptor or SMAD3 have a strong impact on the homeostasis of this tissue [267]. Despite the different disease etiologies, LDS clinical manifestations are related to MFS in the vascular system because of TGF-β signaling pathway dysregulation. LDS results from heterozygous substitutions in crucial residues of the kinase domains of types I or II TGF-β receptor or Smad3, which theoretically abrogates TGFβ signaling. Paradoxically, such signaling remains hyperactivated. Much less is known about LDS in comparison with other syndromes, and even less regarding ROS and oxidative stress contribution to the pathology. Nevertheless, a couple of recent studies provide evidence of their involvement. In a similar study previously carried out in MFS patients, the content of enzymatic and nonenzymatic systems involved in redox stress was measured in plasma and TAA from LDS patients [268]. This group observed a significant reduction of antioxidative stress mechanisms (GSH, antioxidant capacity, glutathione peroxidase, glutathione-S-transferase, catalase, and thioredoxin reductase) accompanied by an increase in both SOD and XO activities. Moreover, *NRF2* expression decreased, which explains the reduced expression or activity of antioxidant enzymes. In addition, reduced mitochondrial respiration was observed in cultured SMC from the mouse model of LDS (*TGFBR1^M318R/+^*). Cultured human fibroblasts from LDS and Marfan patients also showed lower oxygen consumption [269].

##### Arterial Tortuosity Syndrome

Arterial Tortuosity Syndrome (ATS) is a heritable disease characterized by twisting and lengthening of the major arteries, hypermobility of the joints, and laxity of skin [270]. ATS is caused by mutations in SLC2A10, which encodes Glucose Transporter 10 (GLUT10) [271]. In ATS, loss of GLUT10 results in defective collagen and/or elastin. Two models explain the onset and development of the disease: (1) The loss of GLUT10 induces a glucose-dependent increase in TGFβ that stimulates cell proliferation in the vessel wall and (2) GLUT10 transports ascorbate (vitamin C), an essential cofactor for collagen and elastin hydroxylases, into the secretory pathway [272]. Considering the essential connection between ascorbate and the redox state of cells (mainly in fibroblasts), the latter hypothesis acquires more relevance. GLUT10 is highly expressed in VSMC and adipocytes, where it facilitates the transport of the oxidized form of vitamin C (1-dehydroascorbic acid, DHA) into mitochondria, which protects against oxidative stress. The loss of function of GLUT10 in a mutant mouse showed much higher mitochondria-generated ROS levels than wild-type mice [175]. Transcriptomic analysis of cultured skin fibroblasts from ATS patients showed an increase in lipid peroxidation sustained by PPARγ function. The rescue of normal GLUT10 expression normalized redox homeostasis, PPARγ activity, and TGF-β signaling, accompanied by partial ECM reorganization [273]. These works highlight the relevance of vitamin C and ROS in arterial abnormalities.

### 3.3. Proteoglycans and Glycosaminoglycans

Hyaluronic acid (HA) is a widely distributed nonsulfated GAG and a major component of the cartilage extracellular matrix and synovial fluid. An oxidative stress environment of elevated OH^•^ and ONOO^•−^, and increased lipid peroxidation, is associated with HA fragmentation. This effect contributes to the inflammatory response in chondrocytes [274]. In addition, HA can be cleaved by hypochloric acid in autoimmune diseases [275]. HA can also have anti-inflammatory and antioxidative actions in chondrocytes [276] by a mechanism involving AKT–NRF2 axis activation [277] Overall, the role of HA during oxidative and inflammatory damage seems to depend on the size of the HA molecule. High-molecular-weight HA provides tissue integrity and low-molecular-weight HA mediates inflammatory responses [278]. However, ROS are also involved in genetic pathologies that affect the biological cycle of proteoglycans (see below).

#### Mucopolysaccharidoses

Alterations in GAGs’ degradation occur with an intra-lysosomal accumulation of nondegraded products, which cause a group of lysosomal storage disorders called mucopolysaccharidoses (MPS). The degradation of GAGs requires 10 different enzymes that have been widely studied: Five sulfatases, four glycosidases, and one nonhydrolytic transferase. Deficiencies have been found in each of them, resulting in seven MPS that share a series of clinical characteristics, though to varying degrees [279,280].

Typical manifestations include skeletal and joint deformities, dysmorphic facial characteristics, dwarfism, and, depending on type and severity, intellectual disabilities, spinal cord compression, increased intracranial pressure, ocular and hearing impairment, respiratory difficulties, gastrointestinal pathology, and umbilical or inguinal hernias [281,282,283,284]. GAGs are normal components of large vessels and cardiac valves [285,286,287]. Deposition of GAGs occurs in the myocardium, the cardiac valves, and the coronary arteries of all types of MPS, resulting in diffuse narrowing of the epicardial coronary arteries, cardiac valve dysfunction, ventricular hypertrophy, and cardiac failure [288]. The most prominent cardiac manifestation present in 60–90% of patients is progressive cardiac valve pathology. Although it is most prominent in MPS I and MPS II, coronary artery narrowing and/or occlusion has been described in individuals with all types of MPS. Large vessels in patients may show increased wall thickness and may either be narrowed or dilated. Systemic hypertension due to arterial narrowing is common among individuals with MPSI and MPSII. In addition, dilation of the ascending aorta and markedly reduced aortic elasticity has been reported in MPS I. This could be attributed to the downstream effects of GAGs on the assembly of tropoelastin, resulting in elastin that is decreased in content and abnormal in structure [288].

Available evidence on lysosomal diseases shows increased ROS production, dysfunctional mitochondria, aberrant inflammatory and apoptotic signaling, and perturbed calcium homeostasis, among other biochemical alterations [289]. An abnormal accumulation of nondegraded GAGs within the lysosomes leads to ROS increase. Given the acidic interior of lysosomes and abundance of the reducing amino acid cysteine, lysosomes would be a perfect environment to foster Fenton-type reactions, making them unusually sensitive to oxidative stress. The disruption of lysosomes can cause a release of hydrolases, undegraded metabolites and iron into the cytosol, causing cell apoptosis or necrosis, and, finally, tissue injury. Additionally, in a loop process, the release of lysosomal content induces secondary ROS production in cytoplasm, which aggravates the oxidative stress [290,291,292].

The involvement of ROS has been reported in MPS pathology. MPS I patients show high lipid peroxidation levels [293]. Furthermore, MPS II patients show global impairment in redox status, evidenced by an increase in lipids and protein oxidation, as well as alterations in SOD and catalase activities [294]. Accumulation of oxidative products are frequent in MPS IIIB [295]. A reduction of antioxidant defense systems together with oxidative-induced DNA, lipid, and protein damage have been described in MPS IVA disease [296]. Cells exposed to oxidative stress enhance the antioxidant defenses in an attempt to reestablish homeostasis. MPS I mice showed increased carbonyl groups and elevated SOD and CAT activities together with a decrease of thiobarbituric acid-reactive substances, which suggests an exposure to oxidative stress in this model [297]. An increase in oxidative damage-related hallmarks coincides with GAGs’ accumulation as very early events in MPS II mice pathogenesis and precedes glial degeneration, which finally leads to neuronal death. Additionally, the anomalous mitochondrial pattern observed in astrocytes supported the presence of oxidative damage in MPS II progression [298].

An upregulation of NADPH and pro-inflammatory cytokines in MPS IIIB knock-out mice due to microglia activation has been reported [299]. Intense production of superoxide anion, whose main sources during inflammatory conditions are NOXes 1 and 2, enhances the production of other ROS, such as H_2_O_2_ and ONOO^−^, which enhances the oxidant environment [300]. As a result, an increase of ROS and NOS occurs due to microglial Nox and iNOS activation [301,302]. In MPS IIIA mice, a potential link between inflammation and oxidative stress has also been reported [303]. In animal models of MPS VI and VII, an inflammatory process caused by intralysosomal accumulation of GAGs has been postulated, which could trigger the release of cytokines, chemokines, proteases, and NO, leading to apoptosis and connective tissue destruction [304]. Pro-inflammatory cytokines can induce the production of oxidants, prostaglandins, and mitochondrial ROS by macrophages, which contributes to the damage found in MPS patients [305].

Enzyme replacement therapy (ERT) with recombinant human enzymes is a treatment that intends to deliver sufficient enzyme activity to reduce and prevent the accumulation of undegraded substrates. This therapeutic strategy has been used in patients with MPS types I (laronidase), II (idursulfase-α), IVA (elosulfase-α), and VI (galsulfase) [306,307].

MPS IVa patients with ERT presented oxidative and inflammatory imbalance even after eight months of ERT treatment [296]. In MPS II patients, a protective effect against oxidative stress was observed during the first six months of ERT treatment [294]. Nevertheless, even during long-term ERT, some degree of inflammation, oxidative, and nitrative imbalances occur in these patients. These alterations seem to be induced by GAGs’ accumulation and pro-inflammatory cytokines. Notwithstanding, ERT is known to reduce GAGs’ levels and was efficient at improving several biomarkers of oxidative stress [308]. After six months of gene therapy in a mouse model of MPS IIIb, there was a significant reduction in the expression of *Ccl3,* which plays an important role in the macrophage-dependent inflammatory response. There were also reductions in the inflammatory caspase *Casp 4* and in *Cybb* (gp91^phox^), which is a component of the phagocytic enzyme complex NADPH oxidases [309]. Many studies suggest that as a complement of ERT, antioxidant drugs could be candidates to delay disease onset and progression. Neural stem cell cultures of an MPS II mouse model treated with vitamin E triggered full rescue of the phenotype, both of mutant glial and neuronal cells [298]. In fibroblast of MPS III patients, the accumulation of GAGs was partially restored by supplementation with CoQ10 or an antioxidant cocktail (α-tocopherol, *N*-acetylcysteine, and α-lipoic acid). The efficacy varied, depending on the characteristics of each patient, but the results were encouraging [310].

## 4. Concluding Remarks

Redox reactions are necessary for the normal physiology of cells, tissues, and organs. Redox constituents and their products (radical species) have important autocrine- and, probably, paracrine-signaling functions. Radical species generated at cellular level have a great impact on cellular components (lipids, proteins, and nucleic acids), whose functions can significantly change in the short or long term depending on how long the radicals are present in the cell environment. It is evident that when radicals are constantly produced and exceed the buffering capacity of endogenous antioxidants, the physiological role of ROS becomes detrimental, which leads to oxidative stress. In this review, we examined the impact of ROS and oxidative stress in genetic diseases of CT. ROS and oxidative stress are involved in the CT pathology of different genetic diseases at molecular and cellular levels. They have gained relevance in ECM organization and dynamics because ECM (re)modeling is always determinant in the normal homeostasis of the tissue and associated pathologies. Due to increasing awareness of this factor, new therapeutic antioxidant approaches are applied to many of these reported diseases to halt or mitigate clinical symptoms and/or progression of the examined disease. However, this is not easy because oxidative stress can be generated by the dysregulated production of ROS or by dysfunctions of the scavenger systems. In the end, the results might be the same (i.e., oxidative stress), but identification of the precise mechanism by which oxidative stress is generated is essential for a successful therapeutic approach. Further work is necessary to understand the real impact of oxidative stress on the generation and/or progression of genetic diseases (in this case, those affecting connective tissue). Pharmacological interference of oxidative stress in genetic diseases that affect CT formation deserves more attention.

## Figures and Tables

**Table 1:** Summary of the genetic diseases in which connective tissue molecular components are affected, their causative gene with OMIN and ORPHAN numbers, the radical species involved and reported therapeutic approaches.

Molecular Target	Genetic Disease	Gene	Omim Number	Orphan Number	Redox-Associated Dysfunction	Antioxidant or Antioxidant-Related Therapeutics
Collagen Fibers	Alport syndrome	*COL4A3*	203780	88919	Urinary HO-1 and H2O2; mitochondrial ROS; reduction GSH:GSSSG ratio; mitochondrial respiration dysfunction	Anti-miR 21. Osteopontin deficiency
		*COL4A4*	104200	88918		
		*COL4A5*	301050	88917		
	Bethlem myopathy (BMP)	*COL6A1* *COL6A2* *COL6A3*	158810	610	Augmented mitochondrial MAO-induced ROS	Inhibition of cyclophilin D
	Ullrich congenital muscular dystrophy (UCMD)		254090	75840		
	Myosclerosis myopathy (MSMP)		255600	289380		
	Fuchs syndrome (FS)	COL8A2	136800	98974	Overall ROS increase; SOD, catalase, Glutathione peroxidase and reductase depletion; downregulation of peroxiredoxin antioxidants;	Elamitrepide (avoids peroxidation of cardiolipin)
	Collagen XV deficiencies	*COL15A1*	120325 (GEN)	-	Overall ROS increase; mitochondrial dysfunctions;	Cyclosporine A; losartan
Elastic Fibers	Supravalvular aortic stenosis (SAS)	*ELN*	185500	3193	Increased ROS by NCF1 overexpression	
	Williams-Beuren syndrome (WBS)	1.55–1.83 Mb at chromosomal band 7q11.23 deletion (including *ELN*)	194050	904	One copy deletion of *NCF1*; increased superoxide anion; increased nuclear levels of NFR2; mitochondrial dysfunction by DNAJC30 loss; increased aortic nNOS expression and activity	Reduction of Ncf1 expression; apocynin; losartan; epigalloctechnin-3-gallate (EGCG)
	Cutis laxa (CL)	*ELN*	123700	90348		
		*FBLN4*	614437	90349		
		*FBLN5*	219100	90349		
		*LTBP4*	61377	-		
		*ATP7A*	304150	198		
		*ATP6V0A2*	219200	357058		
		*PYCR1*	612940	357064	Mitochondrial antioxidant unbalance	
		*ALDH18A1*	219150	35664		
	Aortic tortuosity syndrome (ATS)	*SLC2A10*	208050	3342	Dehydroascorbate transport dysfunction-induced ROS increase	
	Marfan syndrome (MFS)	*FBN1*	154700	558	Overall increased ROS levels; increased H_2_O_2_ by LOX dysfunction and/or NOX4 overexpression; increased NO breakdown; increased aortic iNOS expression; increased peroxynitrite and 3′-nitrotyrosine residues; glutathione reduction	Losartan; Indometathin; Apocynin; Cobinamide; N-acetylcysteine; Resveratrol;
	Weill-Marchesani syndrome (WMS)	*FBN1*	608328	3449	Increased plasma levels of LPO and NO; reduced antioxidant capabilities;	
	Systemic sclerosis (SSc)	Association with *FBN1*	181750	90291	Increased circulating and tissue ROS levels; NADPH oxidase (NOX2 and NOX4) overexpression	Sindenafil; Kaempferol: EGCG; Tocilizumab; Hydrogen sulfide; Dimethyl fumarate
	Loeys-Dietz syndrome (LDS)	*TGFBR1*	609192	60030	Reduced plasma levels of general antioxidant systems increased SOD and XO activities reduced mitochondrial respiration	
		*TGFBR2*	610168	60030		
		*SMAD3*	613795	91387-284984		
		*TGFB2*	614816	91387		
		*TGFB3*	615582	91387		
Ground Substance	Mucopolysaccharidosis (MPS) I	*IDUA*	607014 60716	579 93473-93476-93474	Overall increased ROS; mitochondrial dysfunction; increased lipid peroxidation; SOD and catalase dysfunctions;	Enzyme replacement therapy.
	MPS II	*IDS*	309900	580 217093-217085	Anomalous mitochondrial pattern	Enzyme replacement therapy. Vitamin E
	MPS III	*SGSH* *NAGLU* *HGSNAT* *GNS*	252900 252920 252930 252940	581 79269 79270 79271 79272	NOX1 and NOX2 upregulation; increased H_2_O_2_ and peroxynitrite;	Coenzyme Q10; Antioxidant cocktail (α-tocopherol, N-acetylcysteine and α-lipoic acid)
	MPS IV	*GALNS* *GLB1*	253000 253010	582 309297 309310	Reduction of antioxidant defense systems together with oxidative-induced DNA, lipid, and protein damage	Enzyme replacement therapy
	MPS VI	*ARSB*	253200	583	release of NO	Enzyme replacement therapy
	MPS VII	*GUSB*	253220	584	release of NO	
